# Perceptions of the Values and Outcomes to Improve Cancer Experiences (VOICE) tool: a digital values-clarification instrument and tailored summary report for older adults with advanced cancer

**DOI:** 10.1186/s41687-026-01038-9

**Published:** 2026-04-02

**Authors:** Amy C. Cole, Angela M. Stover, Lisa Vizer, Elizabeth Kwong, Carl Mhina, Fei Yu, Andy J. King, Lukasz Mazur, Daniel R. Richardson

**Affiliations:** 1 Carolina Health Informatics Program, The University of North Carolina, 321 S. Columbia St, Chapel Hill, NC 27599 USA; 2 Division of Healthcare Engineering, Department of Radiation Oncology, The University of North Carolina, Chapel Hill, NC USA; 3 The University of North Carolina at Chapel Hill Gillings School of Global Public Health, Chapel Hill, NC USA; 4 Lineberger Comprehensive Cancer Center, The University of North Carolina, Chapel Hill, NC USA; 5 Department of Population Health Sciences, Duke University, Durham, NC USA; 6 University of Utah, Huntsman Cancer Institute, Salt Lake City, UT USA

**Keywords:** Values-clarification, Advanced cancer, Best-worst scaling, Tailored health information

## Abstract

**Background:**

Older adults represent the majority of patients with advanced cancer, yet this group frequently reports a lack of discussion of their values as part of treatment decision-making. We previously developed a values-clarification tool called Values and Outcomes to Improve Cancer Experiences (VOICE) that uses best-worst scaling methods to prioritize treatment values and generates a tailored summary report to support patients in talking with their oncologist during treatment decisions. VOICE assesses 7 treatment values: doing activities that are meaningful; managing medical expenses; maintaining independence; minimizing side effects; reducing time spent receiving care; accessing resources for understanding treatment plans; and living longer. The objective of this study was to evaluate older adults’ perceptions of VOICE for comprehensibility and acceptability.

**Methods:**

We used an exploratory sequential mixed-methods study to assess perceptions of VOICE with qualitative (think-aloud protocols and semi-structured interviews) and quantitative (questionnaires) data. Interview questions focused on how older adults may perceive VOICE to affect or prepare them for values-based communication and decision-making with their oncologist. Interviews were coded thematically by three coders, and questionnaires were summarized descriptively.

**Results:**

Fifteen older adults (ages 62–88; 60% female; 60% Caucasian, 27% Black, 13% other) with advanced cancer participated. Participants understood the treatment values as presented in VOICE, and anticipated VOICE having a positive influence on their self-efficacy, confidence, motivation, and likelihood to discuss values with oncologists. Descriptive findings showed that 74% (*n* = 11/15) felt more prepared for meaningful discussions with their oncologist about long-term health status and treatment plans. Participants also perceived that VOICE was helpful in preparing them for discussions with oncologists by helping them to advocate for themselves (*n* = 10/15), self-reflect on what is important when making treatment decisions (*n* = 10/15) and reduce their anxiety (*n* = 3/15). Five interview themes were identified: (i) balancing treatment and everyday life, (ii) patient involvement and value assessment, (iii) emotional and psychological impact of diagnosis and treatment, (iv) communication and relationships, and (v) processing and seeking information.

**Conclusion:**

Older adults with advanced cancer perceived the VOICE values clarification tool to be comprehensible, relevant, acceptable, and useful for improving communication with their oncologist. Further evidence is needed to better understand how patients and oncologists engage in values-clarification processes, and whether implementing tools into clinical practice helps align patient values with treatment recommendations.

## Background

Older adults (≥ 60 years) represent 78% of the US population living with cancer [1], including most advanced stage cancer diagnoses [2], yet are underrepresented in many types of cancer research [3]. Key stakeholders suggests more research is needed to develop patient-clinician communication strategies involving older adults’ perspectives and to use the evidence to inform clinical practice [3, 4]. Developing these communication strategies may overcome the challenge reported by many older adults that the guidance they receive from their oncologist when making treatment decisions does not align with their values [5–11]. Patient “values” are the unique preferences, concerns, and expectations a patient brings to a clinical encounter, which should be integrated into clinical decisions for effective patient care [12]. When patients do not engage in values-based communication with their clinicians, it can lead to decisions that negatively impact their quality of life [13], including undergoing aggressive treatments due to patients’ unrealistic expectations of a cure [9, 14, 15].

Prior studies suggest that dedicated decision support tools including values clarification methods may be useful strategies to clarify patient values [16]. Best-worst scaling (BWS) is a type of values-clarification method that uses a theory-driven approach to prioritize the importance of attributes and has been increasingly used in healthcare to elicit patient values [17–19]. BWS allows for varying data analysis methods, making it a robust method for prioritizing the importance of attributes, and can be easily integrated into digital health tools to facilitate analyzing the data and presenting results [17].

Another communication strategy that may help overcome the reported misalignment between patient and provider values is the use of question prompt lists (QPLs). Oncology-specific QPLs encourage patients to actively participate during consultations, including discussing difficult topics about treatment decision-making [20–23]. QPLs that are tailored to individual patient values are perceived by patients as more relevant and may be more effective at improving communication than static QPLs [24–28].

We previously developed the Values and Outcomes to Improve Cancer Experiences (VOICE) tool, which uses BWS to elicit and prioritize treatment values and generates a tailored summary report that aligns QPLs to patients’ values to support values-based communication between patients and clinicians [29, 30]. In response to calls for improved patient engagement [31] and communication strategies for older adults with cancer [32], this study aims to evaluate how older adults’ perceive the VOICE tool and its potential impact on cancer communication in clinical settings.

## Methods

### Prior work and study materials

To inform the development of VOICE, we conducted a stakeholder-driven concept mapping study involving seven older adults with advanced cancer, two caregivers (not associated with the patients in the study), and twelve oncology specialists [29, 30]. Stakeholders identified seven treatment values through this process, including (a) doing activities that are meaningful to me, (b) managing my medical expenses, (c) maintaining my independence, (d) minimizing side effects, (e) reducing my time spent receiving care, (f) accessing resources for understanding my treatment plans, and (g) living longer. Stakeholders also prioritized 2–4 question prompts aligned to each treatment value, resulting in a total of 24 questions.

**Fig. 1:**
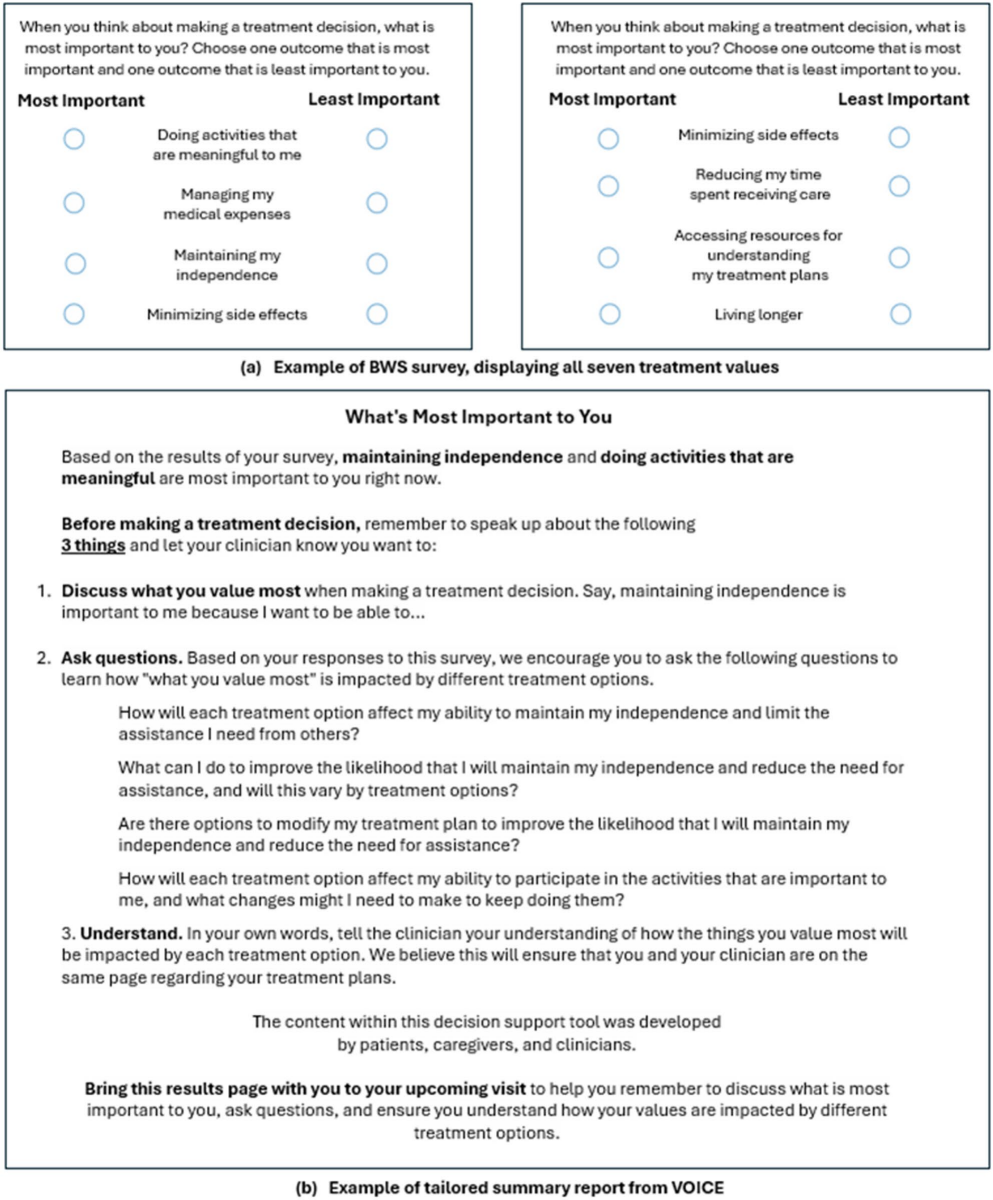
Example of Best-Worst Scaling (BWS) and tailored summary report from VOICE

This work resulted in VOICE, a digital values-clarification instrument that uses Best-Worst Scaling (BWS) to elicit and prioritize a patients values and generates a tailored summary report that includes question prompts aligned to their most important treatment values (See Fig. 1). This foundational work ensured the content of VOICE was grounded in the lived experiences and informational needs of older adults with advanced cancer.

### Study design

We used an exploratory sequential mixed-methods approach in which the qualitative phase served as the primary measure [33]. Using a mixed methods approach allowed us to understand how older adults both perceived the VOICE tool and understood the treatment values presented while capturing complementary descriptive evidence from structured questionnaires. Participants first completed think-aloud protocols and semi-structured interviews to evaluate their perceptions of VOICE, including understanding of treatment values and how the tool might influence their perceived preparation for values-based discussions in clinical settings. After completing the qualitative assessments, participants completed a set of quantitative questionnaires. These questionnaires were used as secondary measures to provide descriptive and complementary evidence regarding their perceptions of effectiveness, relevance, acceptability and preparedness, rather than to produce generalizable conclusions. This approach is appropriate given the small sample size (*N* = 15) and is consistent with mixed-methods usability research where sample sizes of 10–15 participants are typical for think-aloud testing and thematic saturation [34, 35].

We used purposive sampling to recruit older adults with advanced cancer, as this population is the intended user group for VOICE. Inclusion criteria were participants ≥ 60 years of age with a confirmed diagnosis of advanced cancer (metastatic or stage IV), not limited by cancer site. Eligible participants were sequentially approached by study team members either via phone or email. Participating patients gave written consent for this study.

Participants were asked to think aloud while completing the BWS instrument and reviewing the tailored summary report. They engaged in a semi-structured interview (see Appendix A for interview guide), and completed questionnaires to assess perceived effectiveness, relevance, acceptability, and usefulness of VOICE. Participants received $50 for their participation.

Reporting of the mixed‑methods procedures follows GRAMMS (Good Reporting of A Mixed Methods Study) guidelines [36]. (see Appendix B)

This study was conducted between May and June 2024. Sessions were held in a hospital meeting room where VOICE was accessed on iPad or via Zoom through a shared screen. This study was reviewed and approved by the (University of North Carolina at Chapel Hill) (Institutional Review Board Number: 24-1566).

### Data collection

#### Participant characteristics

Participant characteristics included age, sex, race, ethnicity, cancer diagnosis, education level, household income, employment status, and comfort level with technology.

#### Primary measures - qualitative

Participants provided subjective feedback during semi-structured interviews about their perceptions of VOICE, including understanding of treatment values, and whether VOICE impacted their self-efficacy, confidence, motivation or likelihood to-act. (See Appendix A for questions related to these measures) To ensure consistent interpretation of treatment values, we used a process similar to back translation—commonly applied in validating translated assessment tools—to evaluate alignment between participants’ understanding of each value and the original intent as defined by the stakeholder group that developed them [37–39]. This refinement focused on maintaining conceptual clarity and minimizing potential misinterpretation of treatment values by future patients using VOICE. Our approach is supported by best practices in decision support tool development, which emphasize the importance of evaluating user understanding before implementation [40–42].

#### Secondary measures - quantitative

Perceived message effectiveness (PME) was assessed using a modified three-item PME scale [43], originally validated for evaluating health messages in smoking prevention, yet al.so designed for adaptation to other health behaviors. This makes it appropriate for assessing perceived effectiveness. PME items included (i) How much does this report make you believe that sharing your preferences with your oncologist will ‘benefit’ you?, (ii) How much does this report make you think that sharing your preferences is a ‘good idea’?, and (iii) How much does this report ‘encourage’ you to discuss your preferences with your oncologist? Scales range from [1] not at all to [5] a great deal.

Perceived message relevance (PMR) was assessed using a non-validated two-item Likert-type scale [44]. This scale has been applied in earlier studies to assess the personal relevance of tailored health messages, supporting its use in evaluating how well the report in VOICE resonated with participants [44, 45]. PMR Items included (i) The report seemed to be written personally for me, and (ii) The report was relevant to my situation. Scales ranged from [1] completely disagree to [5] completely agree.

Acceptability of Intervention Measure (AIM) was used to assess the acceptability of the report in VOICE. This four-item validated scale has been widely used across health intervention studies and includes (i) the report meets approval, (ii) is appealing, (iii) is liked, and (iv) is welcomed. Scales ranged from [1] completely disagree to [5] completely agree [46].

Preparation for Decision-Making (PrepDM) was assessed using a validated 10-item scale. PrepDM is commonly used to evaluate the usefulness of decision aids in preparing patients for clinical encounters [47]. This measure is appropriate for assessing how well the report in VOICE was perceived to prepare them to discuss their values with their oncologist. Scales ranged from [1] not at all to [5] a great deal.

#### Data analysis

Integration of mixed-methods occurred during data analysis. Data analysis were informed by oncology clinical practice guidelines that emphasize that a patient’s ability to meaningfully participate in decision-making depends on discussing what matters most to them, addressing their emotional concerns and preparing them for discussions that occur within complex and time-limited clinical encounters [48]. Qualitative themes provided the primary insights into participants understanding and experiences with the VOICE tool, while descriptive quantitative findings provided complementary evidence that contextualized and expanded these themes. The research team reviewed qualitative and quantitative data to assess concordance and discordance across methods.

Qualitative analyses were performed in MAXQDA [49] and questionnaires were descriptively summarized in Microsoft Excel.

##### Primary - qualitative

Semi-structured interview data were analyzed subjectively using a hybrid approach to thematic analysis, including three phases to refine and assess the meaningfulness of themes [50]. In phase 1, we categorized data by a priori themes based on interview questions. Interview questions focused on how older adults may perceive VOICE to affect or prepare them for values-based communication and decision-making with their oncologist. In phase 2, one researcher (AC) created the initial *a posteriori* codes. Two other researchers (EK and CM) looked for meanings and patterns in the interviews, assessing initial codes and creating new codes where necessary. Discrepancies were collectively addressed, leading to consensus on codes. In phase 3, we combined a priori and *a posteriori* codes into family codes to structure the findings [50]. All codes underwent a comprehensive review and grouped into overarching themes through discussion among the three researchers (AC, EK, CM). Semi-structured interview data were also summarized through frequency counts to report results.

##### Secondary – quantitative

Descriptive statistics were used to summarize participant responses for PME, PMR, AIM, and PrepDM scores.

PME, PMR and AIM total median and IQR were objectively quantified by averaging the measure’s subscales. Data were not normally distributed, therefore median and interquartile range (IQR) were quantified to represent central tendency and variability of scores. The percentage of participants who rated measures 4 or above on a 1–5 Likert-type scale were reported to highlight the percent expressing high effectiveness, relevance and acceptability of the VOICE tool.

PrepDM total median and IQR were objectively quantified by averaging ten subscales. Items were summed, then divided by 10 to provide a median and IQR. Scores were rescaled to a 0-100 scale by subtracting 1 from the summed score and multiplying by 25. Higher scores represent higher perceived usefulness for preparation in decision making [47].

## Results

Forty older adults with advanced cancer were approached, with a target of enrolling fifteen. Sixteen enrolled, with one no-show, resulting in our target goal of 15 participants. Sessions were held in private rooms at (institution masked for review) (*n* = 4/15) or via Zoom (*n* = 11/15). Differences in how participants engaged in think-aloud or interviews were not observed between mode of participation. Table 1 summarizes participant characteristics.

**Table 1:** Participant characteristics

Characteristic	*N =* 15 (%)
Age, years (y)	
Mean (SD)	70 (6.9%)
Range	62–88
Sex	
Female	9 (60%)
Male	6 (40%)
Race	
American Indian or Alaska Native	1 (7%)
Black or African American	4 (27%)
White or Caucasian	9 (60%)
Prefer not to answer	1 (7%)
Ethnicity	
Not Hispanic/Latino	12 (80%)
Unknown/Prefer not to answer	3 (20%)
Diagnosis*	
Adenocarcinoma	1 (7%)
Bone	2 (13%)
Brain	1 (7%)
Breast	5 (33%)
Hepatocellular carcinoma	1 (7%)
Kidney	2 (13%)
Liver	2 (13%)
Lung	2 (13%)
Merkel cell carcinoma	1 (7%)
Pancreatic	1 (7%)
Prostate	1 (7%)
Renal cell carcinoma	1 (7%)
Education	
Less than high school	
High school graduate (included equivalency)	2 (13%)
Some college, no degree	1 (7%)
Associate’s degree	1 (7%)
Bachelor’s degree	4 (27%)
Graduate or professional degree	6 (40%)
Prefer not to answer	1 (7%)
Household income	
Less than $25,000	2 (13%)
$25,000 to $34,999	1 (7%)
$35,000 to $49,999	1 (7%)
$50,000 to $74,999	2 (13%)
$75,000 to $99,999	2 (13%)
$100,000 to $149,999	4 (27%)
Chose to not answer	3 (20%)
Employment status	
35 h a week or more	1 (7%)
Retired	14 (93%)
Comfort level with technology	
Not comfortable, need support	2 (13%)
Somewhat comfortable, need help getting started	5 (33%)
Very comfortable, little or no help needed	6 (40%)
Prefer not to use technology	1 (7%)
Prefer not to answer	1 (7%)

*Self-reported. Some patients have multiple cancer diagnoses at advanced stage

### Primary outcome – qualitative

Ninety-four *a posteriori* codes were developed from the interviews. These, combined with a priori codes (i.e., understanding, self-efficacy, confidence, motivation and likelihood to act) resulted in 16 subthemes, and five overarching themes: (i) balancing treatment and everyday life, (ii) patient involvement and value assessment, (iii) emotional and psychological impact of diagnosis and treatment, (iv) communication and relationships, and (v) processing and seeking information (see Appendix C for all themes, subthemes, codes and example quotes). Participants frequently discussed experiences with their cancer diagnosis, broadening the thematic analysis.

#### Balancing treatment and everyday life

Five of the seven treatment values coalesced under this theme. See Table 2 for key insights. While some participants have grown more comfortable accepting help from family during treatment, many remain concerned about becoming a burden, impacting ability to maintain independence. Meaningful activities range from physical and social to spiritual, and for some, are disrupted by treatment, yet these impacts are not always discussed with clinicians. Inconsistent interpretations around reducing time in care were discovered. For example, reducing time receiving care was intended to reflect the time burden of care, including travel time, waiting periods, and the number and duration of visits to hospitals and clinics. While side effects are expected, experiences and coping strategies vary. Financial concerns, particularly about the burden on family, were widely shared.

**Table 2:** Key insights regarding balancing treatment and everyday life

Treatment Value	Key Insights
Maintaining independence	Continuing daily activities, not losing their mental or physical functions, not relying on or becoming a burden on others. 35% (*n* = 5/15) have become more comfortable relying on family members for assistance as they advance through cancer treatments but would be less comfortable if they become a burden.
Doing activities that are meaningful to me	Traveling, physical (e.g., hiking, walking, gardening, sexual function), social (e.g., parties, theater, informal gathering with friends), and spiritual (e.g., church, singing, prayer groups) activities that were important to continue participating in. 27% (*n* = 4/15) reported that current treatments impact their ability to do meaningful activities, negatively impacting their quality of life, yet have not discussed with their oncologist.
Reducing time spent receiving care	Limiting travel time to appointments, inefficient clinical processes, coordinating appointments to have fewer clinic days, receiving unnecessary treatments or receiving care at home. However, some interpreted as stopping all treatment or focusing on end-of-life care.
Minimizing side effects	Participants report that side effects are an expected part of treatment. Participants had varying experiences with side effects, reporting on severity (either ability to tolerate or not tolerate), strategies for coping with side effects and the importance of reporting side effects.
Managing medical expenses	Costs of receiving care, insurance coverage, and ability/inability to pay medical bills. Over half of participants (*n* = 8/15) discussed general sentiments about the appalling cost of care, often-overlooked expenses, and concerns for leaving their family with unpaid medical expenses.

#### Emotional and psychological impact of treatment

47% (*n =* 7/15) reported that dealing with physical limitations from either cancer or side effects of treatment makes them feel unmotivated, sad or depressed. 33% (*n =* 5/15) discussed that faith and acceptance of their diagnosis help them deal with uncertainty and maintain a positive outlook.

Participants emphasized the importance of maintaining quality of life, including pursuing meaningful activities, and spending time with family and friends, but not necessarily living longer. Participants consistently defined **living longer** as living as long as possible, regardless of how it impacts quality of life. Most participants reported that while they would like a long life, living longer was not most important to them. When participants ranked living longer as least important, they often apologized or clarified that they were not suicidal or depressed, as they assumed others expected them to prioritize living longer.

#### Communication and relationships

47% (*n =* 7/15) reported having ongoing discussions about their values, while others had not previously thought about discussing their values or felt their oncologist did not have time to discuss. All participants reported the importance of discussing their values, and would discuss, if time allowed. However, from one participant’s experience, “discussing values doesn’t filter down to the real world” (Male [M], 63y), since his oncologist had not previously asked about his values.

Almost half (*n =* 7/15) asked questions, similar to the QPLs, over the course of their treatment, but less so at initial diagnosis. 87% (*n =* 13/15) reported that having a list of relevant questions, prepared ahead of time, could help them discuss their values with their care team.

Participants agreed they would like to explain how they understood their values to be impacted by different treatment options. One articulated “I would give feedback for each one, as far as how I’ve understood it. If it were different, if my oncologist, if she thought I was not on point, then I would be receptive to letting her explain. But I have no problem in saying well with Option A, I understand that…” (F, 77y). Some preferred to rely on their oncologist to explain things, rather than repeating their understanding back to the oncologist because they “clam up” or have uncertainty about the terminology being used (F, 65y).

87% (*n =* 13/15) reported they were likely to discuss their values with their family or caregivers after using VOICE. However, 27% (*n =* 4/15) indicated discussing regardless of VOICE. Some participants (*n =* 2/15) were unlikely to discuss with family because it was too emotional and were concerned about making them sad. The remaining 60% (*n =* 9/15) indicated VOICE would help them discuss their values with family members.

#### Patient involvement and value assessment

Most participants preferred an active role in treatment decisions. Some expressed a desire for an active role, but also stated their oncologist already has their best interest in mind, therefore would rely on oncologist’s guidance rather than discuss their values. Participants (*n =* 2/15) with less active roles, expressed concerns about asking values-related questions, feeling they would be “putting the doctor on the spot” (M, 70y) or avoided discussions about treatment outcomes, for fear the outcomes could not be reached (F, 73y). Both felt more comfortable after using VOICE because the report emphasized the importance of discussing their values related to outcomes.

Participants reported VOICE would help with self-advocacy (*n =* 10/15), self-reflecting on what is most important to them (*n =* 10/15) and reducing their anxiety (*n =* 3/15). Most (*n =* 11/15) perceived VOICE would prepare them to engage in meaningful conversations with their oncologist about their long-term health status and treatment plans. Participants reported the need for dialogue, rather than simply receiving information from their oncologist, and that VOICE gave them permission to talk about their values, and “stay on point” about what’s most important.

67% (*n =* 10/15) and 53% (*n =* 8/15) reported their confidence or motivation was positively impacted by VOICE, respectively. 60% (*n =* 9/15) stated VOICE validated what was most important to them. It impacted motivation because it “encourages people to use their voice, be a voice for themselves to advocate for themselves” (F, 73y). It impacted confidence by “having something written out” (F, 65y) and “knowing that these things are important to me, and this is what I need to focus on when I’m discussing it with my oncologist, so it helps me be able to know what to ask” (F, 77y).

#### Processing and seeking information

When asked to define **accessing resources for understanding my treatment plans**, participants focused on information sources, engagement strategies and comprehension and application of information. They focused on their reliance on others or themselves to seek additional information about diagnoses and treatments.

Participants rely on family, friends, or caregivers (*n =* 7/15), oncologists (*n =* 10/15), or themselves (*n =* 9/15) to seek additional information about diagnoses or treatments. 33% (*n =* 5/15) expressed the importance of seeking second opinions. For some, this meant having access to specialists at other healthcare institutes while maintaining their primary oncologists, and for others it meant transitioning to another oncologist.

20% (*n =* 3/15) prefer to avoid seeking information, prefer not to think about their diagnosis, or prefer to rely solely on their oncologist to be the expert without doing additional reading, because learning more puts them in a negative frame of mind. Others (*n =* 8/15) were interested in seeking as much information as possible, often presenting their findings to their oncologists.

87% (*n =* 13/15) reported they were likely to use VOICE in the future (Table 3). Only 13% (*n =* 2/15) indicated they would not use VOICE to assess their values, either because they already felt comfortable with the values-clarification process, or they felt their values were unlikely to change, making this tool unnecessary. Most (*n =* 10/15) stated the importance of reassessing their values at regular intervals regardless of changes in health status. Some (*n =* 3/15) reported it was important to reassess their values only if changes in their health occurred. Most (*n =* 10/15) reported they were likely to print the report.

**Table 3:** Likelihood to act - discuss, print, or use decision support tool in future

Likelihood to Act	Frequency (%, *n*)
Discuss the summary report with others	
Oncologists	100%, *N =* 15/15
Family members or caregivers	87%, *n =* 13/15
Print report from VOICE	67%, *n =* 10/15
Use VOICE in the future	87%, *n =* 13/15

### Secondary outcome - quantitative

Participants’ perception of message effectiveness, relevance, acceptability, and usefulness is shown in Table 4.

**Table 4:** Descriptive Statistics for Quantitative Measures

	Median (IQR)	(%, *n*) ≥ 4 (range 1–5)
Perceived Message Effectiveness (PME)	5.0 (4.7-5.0)	100%, *n =* 15/15
Perceived Message Relevance (PMR)	5.0 (4.3-5.0)	87%, *n =* 13/15
Acceptability (AIM)	5.0 (4.8-5.0)	100%, *n =* 15/15
Usefulness (PrepDM)	95.0 (87.5–100)	n/a

AIM = Acceptability of Intervention Measure

PrepDM = Preparation for Shared Decision-Making

Participants perceived VOICE to be effective for (i) making them feel that sharing their values with their oncologist would benefit them, (ii) sharing their values with their oncologist is a good idea, and (iii) feeling encouraged to discuss their values with their oncologist. Participants perceived VOICE to be (i) written personally for them and (ii) relevant to their situation. Participants perceived VOICE to be acceptable, based on (i) approval, (ii) appeal, (iii) like, and (iv) welcome. Participants perceived VOICE to be highly useful for preparation in decision making.

## Discussion

### Discussion

This study provides new evidence that older adults support the perceived use of VOICE to inform communication with their oncologist. Older adults not only understand the treatment values presented in VOICE but also anticipated VOICE having a positive influence on their self-efficacy, confidence, motivation and likelihood to act. VOICE was also perceived as effective, relevant, acceptable and useful for preparing older adults to discuss their values in relation to treatment decisions.

A recurring theme among participants reviewing VOICE, was the challenge of “balancing cancer treatments and everyday life”. This challenge is reflected in previous studies on treatment preferences among older adults with cancer, which highlight the importance patients place on their functional outcomes and the benefit-risk trade-offs they are willing to make [51]. While patients focus on tangible outcomes of treatments, less is known about the quality of discussion during clinical encounters to ensure patients’ values and concerns are continuously incorporated into treatment decisions. Notably, 27% of participants in this study reported their current treatment negatively impacted their quality of life but had not communicated this to their oncologist. This highlights the potential role that tools like VOICE have in facilitating earlier and more consistent values-based discussions.

Participants expressed the emotional and psychological burden of treatment decision-making. Many described the strategies they have developed to cope with the uncertainty of treatment decisions. Most expressed a desire to live longer, but not at the expense of their quality of life (e.g. maintaining independence, engaging in meaningful activities, and continuing social activities), which are consistent with the values expressed in prior research with older adults with cancer [51–54]. However, nearly half of participants in this study reported the physical limitations from treatments negatively affect these values, leading to emotional and psychological distress. These findings align with previous research that indicates patients without a strong relationship with their oncologists, may struggle to express their emotional and psychological concerns [55]. Engaging with VOICE may help address some of these challenges by providing patients with an opportunity to reflect on their values in advance of clinical encounters. By prioritizing their values, VOICE has the potential to support patients by preparing them in advance to communicate their concerns that might otherwise remain unspoken.

Participants emphasized the importance of communication and relationships with oncologists. While all participants reported positive relationships with their current oncologist, many shared frustrations with previous oncologists who lacked compassion, rushed through appointments, or did not fully address their concerns. These findings are consistent with previous research [5–11]. However, other research indicates patients who perceive their clinicians as compassionate, regardless of actual appointment length, tend to report reduced depression and increased quality of life [56]. This is further supported by research that suggests patients who perceive clinicians did not rush through the encounter, tend to rate relational communication with their oncologists more positively [56, 57]. This suggests that the perceived quality of the encounter may matter more than its duration. Preparing patients for values-based discussions could potentially improve the quality of these interactions and improve patient outcomes. Findings from this study indicate that participants perceived engaging with VOICE would help facilitate meaningful conversations, enabling older adults to better articulate their values. This was further supported by a secondary descriptive analysis of PrepDM scores, which indicated VOICE would be highly useful for preparing older adults for more focused and relevant values-based discussions. These findings align with calls for more research to develop strategies to improve patient-clinician communication that is grounded in patients’ values [3].

Although participants reported a desire to discuss their values, this study revealed a tension between the desire to actively discuss values and passively trusting their oncologist understands their values when guiding them through the decision-making process. Prior studies suggest that patients who passively trust clinicians, based on clinicians accumulated knowledge of their preferences and values, often report more positive relational communication [57]. This highlights the need for not only establishing but also maintaining an ongoing relationship, so patients and clinicians remain aligned [58], and make adjustments to treatments, if values change over time. This study found preliminary evidence that a lack of values-based discussion can negatively affect patients’ quality of life. VOICE may support patient involvement by prompting patients to identify and reflect on what matters most when making treatment decisions. While more evidence is needed to determine the impact of VOICE on values-clarification processes in clinical settings, these findings highlight its potential to improve patient agency in establishing open, continuous discussions about what matters most to them.

Participants expressed a desire to assess, reassess, and discuss their values. Descriptive quantitative findings support VOICE’s perceived effectiveness, relevance, acceptability and usefulness to achieve that goal, particularly for those hesitant to discuss their values. Many participants felt VOICE validated what was most important to them, positively impacting their perceived likelihood of sharing the tailored summary report with their oncologist. The process of self-reflection prompted by VOICE helped participants identify key discussion points and questions they had not previously considered. This aligns with research that indicates completion of patient-reported health information can encourage self-reflection and prompt meaningful discussion with clinicians [59]. These behaviors, likelihood to act and engagement in self-reflection, are also supported by studies on tailored health information [24–28]. This study did not assess participants behaviors in clinical settings or decision outcomes, however their anticipated intent to use VOICE in the future suggests perceived value in preparing patients to engage in higher quality discussions related to their treatment decisions. Oncology clinical practice guidelines highlight that clinicians are essential partners who should help elicit patients’ values, discuss their concerns, and guide conversations to support decision-making [48]. Although clinicians’ perspectives were not evaluated in this study, their perspectives are essential for the implementation of tools, such as VOICE in clinical practice. Therefore, a logical next step is to evaluate clinicians’ perspectives of VOICE, including perceived time burden and how the tailored report may support value-based discussions in real-world oncology settings.

### Limitations and strengths

Study limitations stem from conducting the study at one institution and the small sample with many cancer types represented. Patients from one healthcare institution, though varying in ages, race, and cancer diagnoses, may not represent the broader population. Nearly all participants in this study were retired, therefore we were unable to capture how employment status may shape balancing treatment and everyday life, such as the need to maintain income or avoid treatment-related side effects that could jeopardize their employment. We prioritized evaluating the effectiveness and relevance of VOICE in English, before employing the rigor required to translate VOICE into other languages. As 10% of the targeted population is Spanish speaking, we anticipate securing adequate resources for effectively translating and ensuring cultural relevance of materials to engage with this patient population in subsequent studies. Our sample size was appropriate for usability testing and qualitative analysis; however, it was too small to perform inferential statistical analysis, which limits our quantitative findings to descriptive summaries and restricts generalizability. Older adults are varied in their comfort level using technology. To mitigate challenges with technology, a member of our research team assisted participants as needed, mostly providing instructions for joining a Zoom session.

Study strengths include flexible options to participate in-person or over Zoom, which broadened recruitment beyond the immediate proximity to (institution masked for review). Members of our research team may have been biased toward study outcomes based on prior work in the development of VOICE. Researcher bias was addressed by having additional coders for qualitative analysis who were not involved in the development of VOICE. The study also directly addresses the call for more research on patient-clinician communication strategies involving patients’ perspectives to inform policy and practice.

## Conclusion

This study demonstrates that older adults perceived VOICE as effective, relevant, acceptable, and useful for preparing them to engage meaningfully in values-based discussions with their oncologists. These data provide a clear proof of principle for evaluating VOICE in future studies in clinical settings.

### Practice implications

This study provides preliminary evidence that patients desire support to clarify their values and discuss them with their oncologist. Patients also desire to work together with their oncologist to align their care with what matters most to them though barriers exist to this process. Practice improvement initiatives in oncology to support values-elicitation, values-clarification, and discussion of patient values may address these desires. VOICE is a promising digital tool to support these efforts by providing a systematic approach for patients to clarify and communicate their values. Integrating the VOICE tool into clinical oncology settings could improve alignment between patients’ values and recommended treatment plans.

## Appendix

Appendix A: Semi-Structured Interview Guide

Appendix B: GRAMMS (Good Reporting of A Mixed Methods Study) guidelines

Appendix C: Thematic Analysis – Themes, Codes and Example Quotes

### Appendix A

LCCC 2110 – IRB 21–0228 – (Advanced Cancer & ≥60 years of age)

Thank you for your time today to test a prototype of VOICE, a values-clarification tool. Today we will be testing one prototype, which may not function as the complete working tool would. While you are testing the prototype, please let us know if you thought something would function differently. It’s all part of the feedback process.

The first thing we want to make sure that you know is that we are testing the prototype and not you. You can’t do anything wrong and please don’t worry about mistakes. And please also don’t worry whether you may hurt our feelings by pointing out problems or errors with our tool. We are conducting this testing to improve upon the prototype, so we need to hear your honest opinions and reactions.

First, I will ask you to read through the introductory text and provide feedback, Next, I will ask you to answer 7 questions. After you complete all 7 questions, you can view a summary of your results. I will ask you to provide feedback after you read the summary report.

If you have any questions during the testing, please just ask them. I may not be able to answer it right away since we are interested in seeing how people use the tool when they don’t have someone next to them to help them. But, when we are done, we will try and answer questions. Please let us know if you need a break at any point as well.

With your permission, we will be audio taping this session to record what is happening during our conversation.

The recordings will help us figure out how to improve the tool since I won’t be able to take a lot of notes while we are doing the testing. The audio recordings and any notes that are taken from today’s session will be kept confidential. You have the right to stop participating in any part of this session at any point.

Do you have any questions before we begin?

You will be asked to answer 7 questions that ask you to select one outcome that is most important and one outcome that is least important to you when making a treatment decision.

Please think aloud as you complete these questions and review the summary report. Once you have reviewed the summary report, please let me know.

#### After viewing their results

(ask patient to answer interview questions before completing the Likert style questions in Qualtrics)

#### Open-ended questions

1. You were presented with a summary report based on your survey responses of “what matter most to you” when making a treatment decision. How did it make you feel to read the results?
  - Can you tell me more about that?
2. Can you tell me how you would use this information.
3. What did you like about the report?
4. What did you not like about the report?
5. Can you describe whether there was any specific information that you found helpful in preparing you to discuss this information with your oncologist?
  - Only ask if nothing in the decision support tool was helpful. Can you describe one thing that would help prepare you to discuss what you value most when making a treatment decision?

#### Self-efficacy, confidence and motivation

6. After reading your report how would you feel about *discussing* this information with your oncologist.
  - Can you tell me more about that.
7. After reading this report how do you feel about *asking questions* related to what is most important to you?
  - Can you tell me more about that.
8. After reading this report how do you feel about stating your *understanding* of how “what you value most” is impacted by different treatment options? For example, how would it make you feel to state in your own words how you understood your values to be impacted by different treatment options.
  - Can you tell me more about that.
9. Do you think having the summary report would impact your confidence in talking to your oncologist about your values?
10. Do you think having the summary report would impact your motivation to discuss your values with your oncologist?

#### Likelihood to act

11. Based on the wording in the report, how likely are you to discuss your values with your oncologist?
  - Very likely, somewhat likely, neutral, somewhat unlikely, very unlikely.
  - You responded “RESPONSE” to this question, could you talk a bit about why you selected that option?
12. Based on the wording in the report, how likely are you to discuss your values with your caregiver/family member?
  - Very likely, somewhat likely, neutral, somewhat unlikely, very unlikely.
  - You responded “RESPONSE” to this question, could you talk a bit about why you selected that option?
13. How likely are you to use VOICE in the future? Can you tell me more about that?
14. Can you share any specific examples of how this summary report impacted your perspective regarding what you would discuss with your oncologist?
15. The final suggestion in this report was to print this and take it with you to an upcoming appointment. Do you think you would do that?

#### In VOICE, you were asked to select items that were most important to you and least important to you. Can you tell me in your own words what each of these items means to you

- Doing activities that are meaningful to me
- Managing my medical expenses
- Maintaining my independence
- Minimizing side effects
- Reducing my time spent receiving care
- Accessing resources for understanding my treatment plans
- Living Longer

**Table 5:** Appendix B - GRAMMS (Good Reporting of A Mixed Methods Study) guidelines

Guideline	Section
Describe the justification for using a mixed methods approach to the research question	Methods – Sect. 2.2 Study Design
Describe the design in terms of the purpose, priority and sequence of methods	Methods – Sect. 2.2 Study Design
Describe each method in terms of sampling, data collection and analysis	Sampling; Methods – Sect. 2.2 Study Design Data Collection and analysis: Sect. 2.3.1–2.3.4
Describe where integration has occurred, how it has occurred and who has participated in it	Data Collection; Sect. 2.3.4 Data Analysis
Describe any limitation of one method associated with the present of the other method	Limitations
Describe any insights gained from mixing or integrating methods	Methods – Sect. 2.2 Study Design

**Table 6:** Appendix C

Theme	Subtheme and Coding	Example Quotes
**Balancing Treatment and Everyday Life**	**Doing activities that are meaningful to me** • Limitation of activities • Social • Spiritual • Physical activities • Travel	*“That means doing what I like to do. I’m very active in church. And if that was taken away from me right now*,* I probably would be a zombie. Because that’s my life. I enjoy it. I mean*,* my husband says*,* I have too many roles. But that’s fine*,* because that’s what I enjoy doing.” (F*,* 77y)* *“I’m retired and have a lot of freedom to do that and so activities that I like is being outside. I’ve always loved to hike and to travel and go places and so being able to hike and even though I can’t do like I used to*,* I put a 35 pound pack on my back and be gone for 10 days or five days or whatever. Well*,* I’ve not been able to try that. I did do two nights last August. But those activities have been very limited since I’ve had this diagnosis*,* but I’m still able to do occasional hiking and I’m still able to get outside every single day*,* which is important to me.” (M*,* 64y)*
	**Managing medical expenses** • Not important or high priority • Co-pays for other medications • Covered by insurance/other means • Appalled at medical expense • Challenges with expenses • No concerns for expenses	*“It’s been helpful to have good insurance. Because when I look at the bills that come in*,* and I look at what the insurance paid*,* and then I look at*,* still look at what I owe*,* and I’m like*,* if I didn’t have insurance*,* I would not be under a doctor. And I can understand those people who don’t have insurance don’t go to doctors. Because it’s just ridiculous.” (F*,* 77y)* *“At first when you start talking about managing medical expenses*,* it really is not at the top of the list*,* but you know when you are constantly going for treatments and scans and you’ve been paying your copays all along. The next thing you know*,* you look and then all of a sudden*,* now it’s like 500 hundred thousand. You start thinking about the quality of life that you’re living and can you maintain that quality of life based on the amount of money that you’re having to put forth towards your treatments and again*,* don’t get me wrong*,* I mean I know how much my treatments cost and all that type of thing*,* and I know how valuable my insurance has been and how valuable the whole staff at UNC’s been to you know*,* not cut corners*,* but to give me better ideas as far as how to combat the finance piece of it*,* but it it’s especially when you’re retired and you’re on a set income like we are. It’s getting to a point where you start feeling it a little bit*,* I mean*,* I don’t go to bed every night and wake up every morning thinking*,* Oh my God*,* how am I going to pay my bills? That’s not what I’m saying*,* but I’m saying I have to really start thinking more about priorities*,* I guess when it comes to spending your money*,* or so it wakes you up to that fact.” (M*,* 70y)*
	**Minimizing Side Effects** • Reporting side effects • Developing strategies to cope with side effects • Understanding of side effects • High tolerance for side effects • Severity of side effects • Side effects expected	*“One of the things that happens when you have cancer*,* at least when I’ve had cancer is that you know*,* all sorts of weird things happen to your body*,* and you know*,* this hurts and this itches. Where under normal circumstances*,* I wouldn’t even give it a second thought*,* but because I’m*,* you know*,* dealing with these toxic drugs*,* iit’s hard to determine what’s important and what isn’t so*,* I guess it’s best to go overboard in terms of reporting stuff.” (M*,* 68y)* *“I know that most medications have some side effects more than others. And some can be debilitating. And some not at all. I want to know that the medications and the treatments that I’m taking are not going to be stopping the quality of my life.” (M*,* 66y)*
	**Reducing Time Spent Receiving Care** • Inefficient processes • Receiving care at home – independence • Unnecessary Treatments • Could not clearly define • Coordinating appointments • Fewer treatments/appointments • Travel time • Terminating Tx/End of Life	*“It means that you getting ready to leave out of here. OK*,* you get ready to check out. All right*,* so I ain’t going to reduce it.” (F*,* 68y)* *“Not going through any unnecessary procedures*,* unnecessary like for example when I went in there and they told me I’m going to need a biopsy. If I was at a major center*,* I would have had a PSMA PET scan that showed everything that they needed to see and find out*,* and plus more*,* as opposed to me getting the biopsy from the urologist.” (M*,* 63y)* *“I don’t want to spend*,* like I said*,* this could change*,* but if I had to go for treatment every day and spend hours at the clinic*,* I just*,* I wouldn’t. I wouldn’t want to do that. You know*,* I want to spend less time there*,* more time at*,* you know*,* at home.” (F*,* 65y)*
	**Maintaining Independence** • Ability to choose therapy • Financially independent • Concerns with losing mental/physical functions • Comfort with relying on others • Continuing day-to-day activities • Not burdening others • Not relying on others	*“When I’m able to contribute to the conversation*,* or the action around me*,* whether that’s in my own place*,* or in the place with someone else*,* that’s as far as I can pay my own way*,* either financially*,* or by being helpful. Pay your own way. That’s being independent.” (F*,* 73y)* *“I don’t want to be a burden on my family. I don’t want to have to depend on someone to bathe me*,* to bring me my meals*,* to cook for me. I mean*,* and they want to do it. But it’s because I’ve done it so long that I just don’t feel comfortable doing it.” (F*,* 77y)* *“I’ve always been very*,* very independent*,* even during marriage and whatever*,* and I just*,* I’ve learned to realize you do need some dependency.” (F*,* 63y)*
**Patient Involvement and Value Assessment**	**Patient Involvement - role of reports** • Indifferent to report • Questions - Helpful • Questions - Not Helpful • Report is educational • Confidence • Patient preparedness • Impacts on Confidence • Motivation • Patient preparedness • Impacts on Motivation • Initial consultation with oncologist • Use of the report in the future • Reduce anxiety • Patient advocacy	*“I think it motivates me because I get a sense of more confidence. I’m understanding her (oncologist)*,* and she’s understanding what I’m trying to ask. Or what questions I may have*,* which may not have come up*,* had I not done this.” (F*,* 77y)* *“I think it’s important that they (oncologist) know what’s important to you. It helps the patient provider relationship when they are willing or*,* they listen. And the only way you know they’re listening is if some of these things that you have said have been incorporated in your care.” (F*,* 65y)* *“I print it (summary report) out and bring in to the doctor and I’ll just highlight what appealed to me most about it or what was more specific to me*,* and I think it’ll really open up conversation with my doctor next time.”* *(F*,* 65y)* *“It’s put into a concise form*,* it gives me a better understanding when I do go in and talk to the provider*,* as far as questions to ask and being able to branch out if there’s other options and that type of thing. I feel more comfortable as far as what to ask.” (M*,* 70y)* *“It might lessen the anxiety*,* you know*,* this is the thing that I experienced*,* is the anxiety*,* and emotional pressure of coming to visit and do lots of tests and things. And then here*,* I’m suddenly presented with information*,* which I need time to process.” (M*,* 88y)* *“And in preparation to talk with the doctor*,* so you can go in fully confident. Because a lot of times you’re not thinking as well because you’re nervous. So I think*,* you know*,* going in to meet and then addressing and outlining things and get into a good place where you feel that they have the best interest and the best possible care you know is important and that’s the best I think the tool can offer*,* helping you relax*,* breathe and know that you have something in hand that will help.” (M*,* 63y)* *“I love this tool. Look*,* it really*,* it really is following me and what I feel. And so I’m just sharing it with you because this is really where I’m at and it just opens up for discussion if you haven’t been able to really have one. Or maybe get somebody to understand.” (F*,* 63y)*
	**Exploring and Understanding Values** • Articulate how they would express their understanding • Did not articulate how they would express their understanding • Comfortable clarifying their understanding • Report not reflection of real-world experience • Doesn’t resonate with treatment values • Validation of Treatment Values • Challenging to select values • Other Most Important Values	*“Choosing between these (treatment values) sometimes is very difficult because they’re intertwined.” (F*,* 74y)* *“The most important is actually kind of tough*,* I think*,* because. UM. I think that there’s kind of two pieces to it. You know there’s two most important. And I think*,* you know*,* one kind of leads to the other. For example. Maintaining my independence*,* I think envelops all three of the remaining items. You know what I mean?” (M*,* 68y)* *“Although I want to pay my medical expenses*,* that’s not that important to me. I mean I like to be able to keep up with them*,* but not really high on the priority list when I look at maintaining me. So that’s my least important. I know it’s easy to find the least important. Now comes the hard part”. (F*,* 77y)* *“I have two most important in my mind and one the least important. I’m gonna go with that on the least important but I kind of like doing activities that are meaningful to me as well as knowing what my options are. I can spend a lot of time thinking about that.” (F*,* 77y)*
	**Patient Roles and Preferences** • Didn’t have treatment options - no need to evaluate • Not ready to evaluate at diagnosis • Role in Treatment Decision Making • Patient Advocacy • No need to assess values again • Assess values only if something change • Assess values at diagnosis • Desire to reassess values regardless of changes	*“I see so often that when you make a treatment plan*,* the uncertainty of what’s going to happen gets lost*,* and it is like you said this was going to happen. And then you’re super disappointed*,* because what you expected to happen*,* didn’t happen. I may not get what I want in the first place so I’m not gonna say what I want. So how do you balance that between expressing what you want and knowing that you may not get it?” (F*,* 73y)* *“Oh yeah. I mean*,* because it’s not them it’s going through it. It’s me*,* so*,* I found that very quickly*,* I have to be my own best advocate to find out what’s going on.” (M*,* 63y)* *“I’m not making those hard decisions. I mean*,* it’s really pretty easy decision making because you’re on the protocol*,* the protocol fails*,* then we go to protocol 2 and I don’t have to worry about what that is*,* because Doctor X knows. And I’m grateful to not have to make those decisions.” (F*,* 73y)* *“You get into the treatment routine*,* but sometimes you don’t get a chance to really stop and think of what my life looks like*,* and what I want my life to look like.” (F*,* 74y)* *“It’s the fact that you might have options that make this important. Even though I did not have a lot of choices to make*,* it would have helped me to go through it.” (F*,* 77y)*
**Emotional and Psychological Impact of Diagnosis and Treatment**	**Reflections on Life and Values** • Putting affairs in order • Quality of life (QOL) • Living longer • Related to good treatment plan • Living longer depends on QOL • Length of life • Reassessing values over time • Spirituality • Self-reflection	*“It’s opened my eyes to things that I’ve always had in the back of my mind that I want to ask but didn’t know how to ask them.” (M*,* 70y)* *“Some of it may be things that I wouldn’t even have thought about asking or not*,* in terms of how I felt. I mean*,* it’s good to know that these things are important to me. And as a result of them being important to me*,* this is what I need to focus on when I’m discussing it with my oncologist. So it helps me be able to know what to ask.” (F*,* 77y)* *“Of course*,* I want to live longer. But I want to for specific reasons. And it’s just good to remember that*,* because I think any treatment plan I take is going to have severe consequences. And*,* yeah*,* I need to remember that.” (F*,* 77y)* *“I mean*,* I think it is certainly we all want to be here as long as we can. But at the same time*,* I would much rather have a high quality of life. I’d rather be able to get out and about and die six months sooner rather than live a year and spend the six months on a in a bed or on a sofa.” (M*,* 68y)* *“I’m not so worried about living the number of days as much as I am about just doing the things that I like to do the best that I could do them with my family. And you know*,* let them know*,* I just put the rest in God’s hands.”* *(F*,* 63y)* *“I understand very well I can stop treatment at any time*,* but you know*,* sort of like the aging process*,* you know*,* you just sort of keep accepting because it keeps changing*,* by golly*,* and there’s not a thing I can do about it. And some days it’s hard to accept that. And some days it’s just how it is and I’m OK with that.” (F*,* 73y)*
	**Emotional well-being** • Anxiety • Positive emotions • Sadness • Depression	*“You go through stages*,* but you know you’re in shock and then you feel sorry for yourself and you know all that stuff.” (M*,* 70y)* *“I’ve actually been blessed in lots of ways with this diagnosis*,* one way is that I’ve been able to have the time to prepare and do the things that I needed to do that I had done far as my end of life goes.” (M*,* 64y)* *Trying to do activities with those treatment. And treatment limits you so much and it makes you feel like you don’t want to do anything*,* you know. You know*,* it can get you in a state of depression.” (M*,* 63y)*
**Communication and Relationships**	**Communication and Information Sharing** • Interest in having a copy of summary report • Had not previously been discussed, prompted discussion • Pre-tendency to discuss • Share information with others (oncologist, family, etc.)	*“I’d like to have it (summary report) so I could review it again before I go into the doctor and then after we go over everything you know*,* then I can say. Doc*,* let me let me ask you a few questions that I haven’t asked in the past that.” (M*,* 70y)* *“I mean*,* I want to know*,* if I’m riding in my car*,* that the odds are high that I may have a need for a bathroom*,* like almost immediately*,* you know*,* that’s pretty important to me. So that’s just a sort of*,* you know*,* an example. But. Yeah*,* I would definitely like to pin the oncologist down about all of those (questions from summary report)*,* and make sure that I was clear on what their instructions were to me. So this is a nice little summary of*,* you know things to be especially keen on.” (M*,* 68y)* *“[I]t would be important for me to know*,* how it would limit activities or what changes in my lifestyle it would cause.” (F*,* 77y)* *“It addresses*,* you know*,* issues that are relevant to me.” (F*,* 65y)*
	**Family Discussions** • Impact on family • Willingness to discuss	*“We don’t pay attention to the elephant in the room*,* we just live our lives around the elephant. We don’t even talk about the treatment anymore. But we really don’t talk*,* we do the things we’ve been doing*,* we seldom talk about our health issues.” (F*,* 73y)* *“You know something*,* they don’t ask me. But my sister*,* with what I’m going through it*,* they the ones crying*,* not me.” (F*,* 68y)* *“Frankly*,* it would be a little harder*,* perhaps because you know the family is a whole different set of ears and understanding their level of understanding. So*,* I mean*,* I would still do it. I think it’s important that there be communication in the family. The only excuse that I’ve given in the past for withholding information was to not burden my wife. I have withheld information*,* which I don’t think is a good thing to do*,* by the way. So*,* in retrospect now*,* and with the benefit of this (summary report)*,* I would be much more likely to be totally upfront.” (M*,* 68y)* *“You know*,* I’ve been open with them (family) from day one. This is like I said*,* pretty much standard for the way I think*,* but it would help other people seeing this (summary report)” * *(M*,* 64y)* *“Because what impacts me impacts them. And so it would be*,* you know*,* hard UM*,* actually not to discuss it with them*,* because I have to depend on them*,* for the support and everything. And so*,* I don’t try to keep anything from my family. So*,* if it concerns me*,* it concerns them.” (F*,* 65y)*
	**Oncologist Relationship** • Trust in oncologist • Quality of relationship • Oncologist relationship - negative • Opposing ideas or discordance • Does not listen • Lack of time • Oncologist relationship - positive • Provides ample time • Listens well	*“Accessing resources is least important to me because I have a lot of faith in my team.” (M*,* 64y)* *“I just started this chemo and she said she’ll call me Thursday or Friday and see how I’m doing. And she does. They follow up. The care team that I have is excellent.” (F*,* 63y)* “*Yes*,* and let me add one other thing. My experience again*,* that’s all I can report on is that like the regular oncologist people that you know*,* the treating doctors*,* I guess you would say those that are prescribing various drugs and or therapies they have kind of their road map for how they need to handle the patient and then the palliative care doctors*,* for example*,* they have a different road map and I think they have more interest and awareness of these kinds of things*,* whereas the mainstream oncology people I think are very focused on treating and so I don’t know. Honestly*,* I don’t know how much they care about our values. You know what I mean? They want to be successful. I think you know they all want to be as helpful as they can*,* but at the same time*,* they’re all rushed for time and they don’t have the time to sit around and have a*,* shall we say*,* a philosophical discussion about what’s most important. I mean*,* they’ve got 30 minutes to see you*,* and then it’s on to the next patient. So*,* we have a lot of ground to cover in 30 minutes. So*,* if I could add something to the whole process*,* it would be to give these oncology people more time.” (M*,* 68y)* *“I mean*,* I have no*,* no qualms at all about speaking up and speaking out about*,* you know*,* all of that (referring to values). I think that I’ve mostly*,* you know*,* my experience has been that some doctors have more empathy and compassion than others. And so*,* you know*,* by and large*,* most of my care providers at UNC are in the sort of outstanding category and they are very very much aware. I think that they’re pretty good about explaining the things*,*” (M*,* 68y)*
**Processing and Seeking Information**	**Comprehension and Application of Information** • Understanding of diagnosis, treatment options, treatment decisions • Summary report questions - Have previously asked these types of question • Reminders • Making future decisions	*“When I was getting immunotherapy they would say*,* oh*,* you’re stable*,* you’re stable. I’d even got something from Dr. X that says you are stable with exclamation points*,* you know. And then that’s what made me think*,* is stable good? Because if you know somebody in the hospital that’s stable*,* they’re not good or bad*,* they’re just okay*,* waiting to see what happens*,* right? But stable in oncology means better. So*,* they really should tell their patients that when they start because I didn’t know up until they told me*,* we don’t detect any cancer in your body any longer. Up until then*,* I thought I was dying. I thought I didn’t know I was getting better.” (F*,* 65y)* *“I think I have a pretty good handle on all of this. I think it certainly doesn’t hurt to be reminded of*,* you know*,* discuss what you value most. It doesn’t hurt to be reminded to ask questions*,* but I think that that’s almost*,* at least for me*,* it’s sort of intuitive to the whole process. Do you know what I mean?”* *(M*,* 68y)*
	**Information Engagement Strategies** • Monitoring (information seeking) • Blunting (information avoiding)	*“I’m extremely inquisitive*,* you know. I think more so than a lot of people. Like*,* I’ll read all of those*,* you know*,* very dry*,* lengthy drug studies. And look for things like treatment related adverse events and the percentages and what they are. That old saying*,* knowledge is power*,* you know*,* I’m a big believer in that. And so*,* I want to know as much about what it is that they’re doing as possible.” (M*,* 68y)* *I like to do research*,* so whenever something is suggested*,* treatment or whatever*,* I have to go and do my own research. I mean*,* I get the literature that’s handed to me and I read through it and then I go and do*,* you know*,* I do my own research*,* you know*,* look up if there’s like words in there that are not familiar to me*,* I go look them up and break it down. So that I understand clearly what’s*,* what all this entails. So I don’t enter into anything blindly.”* *(F*,* 65y)* *“Sometimes I if I read*,* I say I’m not reading it (referring to materials that oncologist gives to her) because it might make me depressed.” (F*,* 68y)* *“I don’t read up on it a lot. Maybe I should read up on it more. I’m not going to do anything that would undermine their treatment for me. I feel comfortable with them without doing any excess reading. And that might not be good*,* but that’s just my philosophy.” (M*,* 70y)*
	**Information Sources** • Relies on family/friend/caregiver • Seeks information independently • Relies on oncologists • Sought second opinion/importance of second opinion • Oncologists providing information/resources	*“Finding more information on my treatment plans*,* finding information. And I can get that from my sister and from my niece*,* some of it for my doctor*,* and some of it I just have to wade through on my own of whether I really want that. Meaning*,* how many treatments do I want.” (F*,* 74y)* *“Being able to look up your medications*,* look up your side effects and look up what the most likely treatment plan is for this given disease process. It doesn’t mean looking up your prognosis of disease. Are we doing the standard of care? And are these side effects to be expected*,* you know*,* is the medication giving me these things that I’m having as it related to my medication*,* that I’m taking. Those are the resources that I need*,* and*,* they pretty much have them available. And those are the things you trust in your physician for if you don’t find something that you need to know*,* you trust your physician to get you that information. And*,* you know*,* I think we’ve got a good team. And what she doesn’t know she’ll send me to somebody who does.” (F*,* 73y)* *“That’s what he’s there for*,* to*,* to not only provide you options*,* but you know*,* address every question about you. Yeah*,* and know what your priorities are and what you feel is where you want to get to and if it’s possible with your current condition. So I mean*,* if he can’t address that*,* then probably need to move on to another oncologist.” (M*,* 63y)* *“And then through discussion I requested*,* you know*,* they suggested I even go to Dr. X in Seattle. And they contacted Dr. X*,* and consulting with the cancer hospital and suggested a treatment.” (M*,* 88y)*

## Data Availability

The datasets used and/or analyzed during the current study are available from the corresponding author on reasonable request.

## References

[1] American Cancer Society (2022) American Cancer Society. Cancer Treatment & Survivorship Facts & Figs. 2022–2024. American Cancer Society, Atlanta

[2] National Cancer Institute Advanced Cancer - NCI [Internet]. [cited 2023 Aug 19]. Available from: https://www.cancer.gov/about-cancer/advanced-cancer

[3] Mollica MA, Smith AW, Tonorezos E, Castro K, Filipski KK, Guida J et al (2022) Survivorship for individuals living with advanced and metastatic cancers: national cancer institute meeting report. J Natl Cancer Inst 114(4):489–495 34878107 10.1093/jnci/djab223 PMC9002286

[4] Gilligan T, Coyle N, Frankel RM, Berry DL, Bohlke K, Epstein RM et al (2017) Patient-Clinician Communication: American Society of Clinical Oncology Consensus Guideline. J Clin Oncol 35(31):3618–3632 28892432 10.1200/JCO.2017.75.2311

[5] Zikmund-Fisher BJ, Couper MP, Singer E, Ubel PA, Ziniel S, Fowler FJ et al (2010) Deficits and variations in patients’ experience with making 9 common medical decisions: the DECISIONS survey. Med Decis Mak 30(5 Suppl):85S–95S 10.1177/0272989X10380466 20881157

[6] Loh KP, Xu H, Back A, Duberstein PR, Gupta Mohile S, Epstein R et al (2020) Patient-hematologist discordance in perceived chance of cure in hematologic malignancies: A multicenter study. Cancer 126(6):1306–1314 31809566 10.1002/cncr.32656 PMC7050385

[7] LeBlanc TW, Fish LJ, Bloom CT, El-Jawahri A, Davis DM, Locke SC et al (2017) Patient experiences of acute myeloid leukemia: A qualitative study about diagnosis, illness understanding, and treatment decision-making. Psychooncology 26(12):2063–2068 27862591 10.1002/pon.4309

[8] Harrison M, Milbers K, Hudson M, Bansback N (2017) Do patients and health care providers have discordant preferences about which aspects of treatments matter most? Evidence from a systematic review of discrete choice experiments. BMJ Open 7(5):e014719 28515194 10.1136/bmjopen-2016-014719 PMC5623426

[9] El-Jawahri A, Nelson-Lowe M, VanDusen H, Traeger L, Abel GA, Greer JA et al (2019) Patient-Clinician Discordance in Perceptions of Treatment Risks and Benefits in Older Patients with Acute Myeloid Leukemia. Oncologist 24(2):247–254 30139841 10.1634/theoncologist.2018-0317 PMC6369944

[10] Beaussant Y, Mathieu-Nicot F, Pazart L, Tournigand C, Daneault S, Cretin E et al (2015) Is shared decision-making vanishing at the end-of-life? A descriptive and qualitative study of advanced cancer patients’ involvement in specific therapies decision-making. BMC Palliat Care 14:61 26572617 10.1186/s12904-015-0057-4 PMC5477801

[11] Mohile SG, Dale W, Somerfield MR, Schonberg MA, Boyd CM, Burhenn PS et al (2018) Practical assessment and management of vulnerabilities in older patients receiving chemotherapy: ASCO guideline for geriatric oncology. J Clin Oncol 36(22):2326–2347 29782209 10.1200/JCO.2018.78.8687 PMC6063790

[12] Institute of Medicine (2001) Crossing the Quality Chasm: A New Health System for the 21st Century. National Academies, Washington, DC 25057539

[13] Chandwani KD, Zhao F, Morrow GR, Deshields TL, Minasian LM, Manola J et al (2017) Lack of Patient-Clinician Concordance in Cancer Patients: Its Relation With Patient Variables. J Pain Symptom Manage 53(6):988–998 28185892 10.1016/j.jpainsymman.2016.12.347 PMC5474148

[14] Back AL, Fromme EK, Meier DE (2019) Training Clinicians with Communication Skills Needed to Match Medical Treatments to Patient Values. J Am Geriatr Soc 67(S2):S435–S441 31074864 10.1111/jgs.15709

[15] Weeks JC, Catalano PJ, Cronin A, Finkelman MD, Mack JW, Keating NL et al (2012) Patients’ expectations about effects of chemotherapy for advanced cancer. N Engl J Med 367(17):1616–1625 23094723 10.1056/NEJMoa1204410 PMC3613151

[16] Witteman HO, Ndjaboue R, Vaisson G, Dansokho SC, Arnold B, Bridges JFP et al (2021) Clarifying Values: An Updated and Expanded Systematic Review and Meta-Analysis. Med Decis Mak 41(7):801–820 10.1177/0272989X211037946 PMC8482297 34565196

[17] Reed Johnson F, Lancsar E, Marshall D, Kilambi V, Mühlbacher A, Regier DA et al (2013) Constructing experimental designs for discrete-choice experiments: report of the ISPOR Conjoint Analysis Experimental Design Good Research Practices Task Force. Value Health 16(1):3–13 23337210 10.1016/j.jval.2012.08.2223

[18] Marley AAJ, Louviere JJ (2005) Some probabilistic models of best, worst, and best–worst choices. J Math Psychol 49(6):464–480

[19] Hollin IL, Paskett J, Schuster ALR, Crossnohere NL, Bridges JFP (2022) Best-Worst Scaling and the Prioritization of Objects in Health: A Systematic Review. PharmacoEconomics 40(9):883–899 35838889 10.1007/s40273-022-01167-1 PMC9363399

[20] Terrasson J, Rault A, Dolbeault S, Brédart A (2022) Question prompt lists to improve communication between cancer patients and healthcare professionals. Curr Opin Oncol 10.1097/CCO.0000000000000840 35730518

[21] Sansoni JE, Grootemaat P, Duncan C (2015) Question Prompt Lists in health consultations: A review. Patient Educ Couns 10.1016/j.pec.2015.05.015 26104993

[22] Clayton J, Butow P, Tattersall M, Chye R, Noel M, Davis JM et al (2003) Asking questions can help: development and preliminary evaluation of a question prompt list for palliative care patients. Br J Cancer 89(11):2069–2077 14647140 10.1038/sj.bjc.6601380 PMC2376858

[23] Keinki C, Momberg A, Clauß K, Bozkurt G, Hertel E, Freuding M et al (2021) Effect of question prompt lists for cancer patients on communication and mental health outcomes-A systematic review. Patient Educ Couns 104(6):1335–1346 33593643 10.1016/j.pec.2021.01.012

[24] Petty RE, Cacioppo JT (1986) The Elaboration Likelihood Model of Persuasion. Elsevier, pp 123–205

[25] de Nooijer J, Lechner L, de Vries H (2002) Tailored versus general information on early detection of cancer: a comparison of the reactions of Dutch adults and the impact on attitudes and behaviors. Health Educ Res 17(2):239–252 12036238 10.1093/her/17.2.239

[26] Brug J, Steenhuis I, van Assema P, de Vries H (1996) The impact of a computer-tailored nutrition intervention. Prev Med 25(3):236–242 8781000 10.1006/pmed.1996.0052

[27] Tawfik E, Ghallab E, Moustafa A (2023) A nurse versus a chatbot – the effect of an empowerment program on chemotherapy-related side effects and the self-care behaviors of women living with breast Cancer: a randomized controlled trial. BMC Nurs 22(1):102 37024875 10.1186/s12912-023-01243-7 PMC10077642

[28] Bull FC, Kreuter MW, Scharff DP (1999) Effects of tailored, personalized and general health messages on physical activity. Patient Educ Couns 36(2):181–192 10223022 10.1016/s0738-3991(98)00134-7

[29] Cole AC, King AJ, Vizer L, Mazur L, Richardson DR (2025) Participatory Engagement and Concept Mapping to Develop a Values- Clarification Tool for Adults with Advanced Cancers. MDM 45(5)

[30] Cole AC, Stover AM, Vizer L, Yu F, King AJ, Mazur L et al (2025) Identifying and prioritizing treatment values and tailored question prompts for older adults with advanced cancer: A concept mapping study. J Geriatr Oncol 16(8):102717 40987198 10.1016/j.jgo.2025.102717

[31] Murphy A, Bere N, Vamvakas S, Mavris M (2021) The added value of patient engagement in early dialogue at EMA: scientific advice as a case study. Front Med (Lausanne) 8:811855 35127766 10.3389/fmed.2021.811855 PMC8811124

[32] M. R, L. R. Patient-Centered Communication in Cancer Care: Promoting Healing and Reducing Suffering. National Cancer Institute (2007) NIH Publication 07–6225

[33] Morse JM (2016) Mixed Method Design. Taylor & Francis Group

[34] Sauro J 3 Ways to Combine Quantitative and Qualitative Research – MeasuringU [Internet]. (2015) [cited 2026 Feb 6]. Available from: https://measuringu.com/mixing-methods/

[35] Nielsen J (1994) Estimating the number of subjects needed for a thinking aloud test. Int J Hum Comput Stud 41(3):385–397

[36] O’Cathain A, Murphy E, Nicholl J (2008) The quality of mixed methods studies in health services research. J Health Serv Res Policy 13(2):92–98 18416914 10.1258/jhsrp.2007.007074

[37] Meireles R, Tomé G, Pinheiro S, Diogo C (2022) BREAST-Q Translation and Linguistic Validation to European Portuguese. Acta Med Port 35(11):823–829 35791701 10.20344/amp.17427

[38] Waghmare CM, Aggarwal VV, Bhanu A, Ravichandran M (2023) Marathi translation, linguistic validation, and cross-cultural adaptation of M.D. Anderson dysphagia inventory in patients of head and neck squamous cell cancer. Indian J Cancer 60(2):199–205 37530241 10.4103/ijc.IJC_1006_20

[39] Alahmadi S, Barata Herrera DM, Heron MJ, Gomez-Rexrode AE, Rivera Perla KM, Soto E et al (2025) Spanish Translation and Validation of the LIMB-Q: A Patient-reported Outcome Measure for Lower Extremity Trauma. Plast Reconstr Surg Glob Open 13(2):e6511 39911535 10.1097/GOX.0000000000006511 PMC11798375

[40] Erturkmen GBL, Juul NK, Redondo IE, Gil AO, Berastegui DV, de Manuel E et al (2024) Design, implementation and usability analysis of patient empowerment in ADLIFE project via patient reported outcome measures and shared decision making. BMC Med Inf Decis Mak 24(1):185 10.1186/s12911-024-02588-y PMC11212241 38943152

[41] Schaaf J, Weber T, von Wagner M, Stephan C, Köhler SM, Voigt A et al (2024) Exploring patient-centered design solutions of a telehealth app for HIV - A qualitative study. Int J Med Inf 189:105524 10.1016/j.ijmedinf.2024.105524 38889535

[42] Gomaa S, Posey J, Bashir B, Basu Mallick A, Vanderklok E, Schnoll M et al (2023) Feasibility of a Text Messaging-Integrated and Chatbot-Interfaced Self-Management Program for Symptom Control in Patients With Gastrointestinal Cancer Undergoing Chemotherapy: Pilot Mixed Methods Study. JMIR Formativ Res 7:e46128 10.2196/46128 PMC10674151 37948108

[43] Baig SA, Noar SM, Gottfredson NC, Boynton MH, Ribisl KM, Brewer NT (2019) UNC perceived message effectiveness: validation of a brief scale. Ann Behav Med 53(8):732–742 30321252 10.1093/abm/kay080 PMC6636889

[44] Jensen JD, King AJ, Carcioppolo N, Davis L (2012) Why are tailored messages more effective? A multiple mediation analysis of a breast cancer screening intervention. J Commun 62(5):851–868 26405350 10.1111/j.1460-2466.2012.01668.x PMC4578294

[45] Cole AC, Kwong E, Mhina C, King AJ, Mazur L, Richardson DR (2024) Perceptions on tailored messages from a values clarification tool: a mixed-methods study of older adults with cancer. Front Commun 9

[46] Weiner BJ, Lewis CC, Stanick C, Powell BJ, Dorsey CN, Clary AS et al (2017) Psychometric assessment of three newly developed implementation outcome measures. Implement Sci 12(1):108 28851459 10.1186/s13012-017-0635-3 PMC5576104

[47] Bennett C, Graham ID, Kristjansson E, Kearing SA, Clay KF, O’Connor AM (2010) Validation of a preparation for decision making scale. Patient Educ Couns 78(1):130–133 19560303 10.1016/j.pec.2009.05.012

[48] Stiefel F, Bourquin C, Salmon P, Achtari Jeanneret L, Dauchy S, Ernstmann N et al (2024) Communication and support of patients and caregivers in chronic cancer care: ESMO Clinical Practice Guideline. ESMO Open 9(7):103496 39089769 10.1016/j.esmoop.2024.103496 PMC11360426

[49] MAXQDA 2022 [computer software] VSoftware (2021) VERBI Software. (2021). MAXQDA 2022 [computer software]. Berlin, Germany: VERBI Software. Available from maxqda.com. Berlin, Germany: VERBI Software. Available from maxqda.com 2022

[50] Swain J (2018) A hybrid approach to thematic analysis in qualitative research: using a practical example. 1 Oliver’s Yard, 55 City Road, London EC1Y 1SP United Kingdom. SAGE Publications Ltd

[51] Seghers PALN, Wiersma A, Festen S, Stegmann ME, Soubeyran P, Rostoft S et al (2022) Patient Preferences for Treatment Outcomes in Oncology with a Focus on the Older Patient-A Systematic Review. Cancers (Basel). ;14(5) 10.3390/cancers14051147 PMC8909757 35267455

[52] Richardson DR, Oakes AH, Crossnohere NL, Rathsmill G, Reinhart C, O’Donoghue B et al (2021) Prioritizing the worries of AML patients: Quantifying patient experience using best-worst scaling. Psychooncology 30(7):1104–1111 33544421 10.1002/pon.5652 PMC10246445

[53] Cole A, Khasawneh A, Adapa K, Mazur L, Richardson DR (2022) Development of an Electronic Healthcare Tool to Elicit Patient Preferences in Older Adults Diagnosed with Hematologic Malignancies. In: Gao Q, Zhou J, editors. Human aspects of IT for the aged population technology in everyday living: 8th international conference, ITAP 2022, held as part of the 24th HCI international conference, HCII 2022, virtual event, june 26 – july 1, 2022, proceedings, part II. Cham: Springer International Publishing 210–28

[54] Nguyen AC, Amspoker AB, Karel M, Stevenson A, Naik AD, Moye J (2023) The what matters most survey: A measurement evaluation of a self-reported patient values elicitation tool among cancer survivors. Patient Educ Couns 115:107899 37467595 10.1016/j.pec.2023.107899 PMC11457758

[55] Step MM, Rose JH, Albert JM, Cheruvu VK, Siminoff LA (2009) Modeling patient-centered communication: oncologist relational communication and patient communication involvement in breast cancer adjuvant therapy decision-making. Patient Educ Couns 77(3):369–378 19811883 10.1016/j.pec.2009.09.010 PMC2787652

[56] Neumann M, Wirtz M, Bollschweiler E, Mercer SW, Warm M, Wolf J et al (2007) Determinants and patient-reported long-term outcomes of physician empathy in oncology: a structural equation modelling approach. Patient Educ Couns 69(1–3):63–75 17851016 10.1016/j.pec.2007.07.003

[57] Shay LA, Dumenci L, Siminoff LA, Flocke SA, Lafata JE (2012) Factors associated with patient reports of positive physician relational communication. Patient Educ Couns 89(1):96–101 22554386 10.1016/j.pec.2012.04.003 PMC3431455

[58] Waldron T, Carr T, McMullen L, Westhorp G, Duncan V, Neufeld S-M et al (2020) Development of a program theory for shared decision-making: a realist synthesis. BMC Health Serv Res 20(1):59 31973754 10.1186/s12913-019-4649-1 PMC6979294

[59] Greenhalgh J, Gooding K, Gibbons E, Dalkin S, Wright J, Valderas J et al (2018) How do patient reported outcome measures (PROMs) support clinician-patient communication and patient care? A realist synthesis. J Patient Rep Outcomes 2:42 30294712 10.1186/s41687-018-0061-6 PMC6153194

